# FOXO3a regulates rhinovirus-induced innate immune responses in airway epithelial cells

**DOI:** 10.1038/s41598-019-54567-3

**Published:** 2019-12-03

**Authors:** Joao Gimenes-Junior, Nicole Owuar, Hymavathi Reddy Vari, Wuyan Li, Nathaniel Xander, Sudhir Kotnala, Uma S. Sajjan

**Affiliations:** 1 Department of Thoracic Surgery and Medicine, Temple University, Philadelphia, PA USA; 2 Department of Physiology, Temple University, Philadelphia, PA USA

**Keywords:** RIG-I-like receptors, Viral host response

## Abstract

Forkhead transcription factor class O (FOXO)3a, which plays a critical role in a wide variety of cellular processes, was also found to regulate cell-type-specific antiviral responses. Airway epithelial cells express FOXO3a and play an important role in clearing rhinovirus (RV) by mounting antiviral type I and type III interferon (IFN) responses. To elucidate the role of FOXO3a in regulating antiviral responses, we generated airway epithelial cell-specific Foxo3a knockout (Scga1b1-Foxo3a−/−) mice and a stable FOXO3a knockout human airway epithelial cell line. Compared to wild-type, Scga1b1-Foxo3a−/− mice show reduced IFN-α, IFN-β, IFN-λ2/3 in response to challenge with RV or double-stranded (ds)RNA mimic, Poly Inosinic-polycytidylic acid (Poly I:C) indicating defective dsRNA receptor signaling. RV-infected Scga1b1-Foxo3a−/− mice also show viral persistence, enhanced lung inflammation and elevated pro-inflammatory cytokine levels. FOXO3a K/O airway epithelial cells show attenuated IFN responses to RV infection and this was associated with conformational change in mitochondrial antiviral signaling protein (MAVS) but not with a reduction in the expression of dsRNA receptors under unstimulated conditions. Pretreatment with MitoTEMPO, a mitochondrial-specific antioxidant corrects MAVS conformation and restores antiviral IFN responses to subsequent RV infection in FOXO3a K/O cells. Inhibition of oxidative stress also reduces pro-inflammatory cytokine responses to RV in FOXO3a K/O cells. Together, our results indicate that FOXO3a plays a critical role in regulating antiviral responses as well as limiting pro-inflammatory cytokine expression. Based on these results, we conclude that FOXO3a contributes to optimal viral clearance and prevents excessive lung inflammation following RV infection.

## Introduction

Members of the forkhead transcription factor class O (FOXO) family are expressed in nearly all tissues and modulate a wide variety of cellular processes with significant implications for health and disease^1–6^. These cellular processes include cellular metabolism, proliferation, apoptosis, DNA repair and resistance to oxidative stress. In the lungs, FOXO3a is expressed in the airway epithelium in addition to macrophages and other cell types^7,8^. In airway epithelial cells,  FOXO3a plays an important role in regulating innate immune responses to infections. For example, FOXO3a was shown to contribute to the expression of antimicrobial factor (human β-defensin 2) and cytokines (IL-6, IL-8, and IP-10) in response to bacterial infection in bronchial epithelial cells^9^. FOXO3a was also demonstrated to positively regulate Poly Inosinic-polycytidylic acid (Poly I:C), an agonist of double-stranded (ds) RNA receptors-induced CXCL-10 in bronchial epithelial cells^9^. In contrast, FOXO3a was shown to negatively regulate virus-induced type I interferon (IFN) expression in macrophages by targeting IFN regulatory factor (IRF)7^10,11^ and in fibroblasts by acting as gene repressor^12^. Since RNA viruses including rhinovirus (RV), induce IFNs via dsRNA, which is generated during viral replication^13^, we hypothesized that FOXO3a may regulate RV-stimulated innate immune responses that are cell type-specific.

FOXO3a plays an important role in protection against oxidative stress by inducing expression of antioxidant enzymes, such as catalase, peroxiredoxin3, and superoxide dismutase^14–16^. Therefore, oxidative stress can potentially increase in the absence of FOXO3a. Previously, we demonstrated that excessive oxidative stress attenuates type I and type III IFN responses to RV infection in airway epithelial cells^17^. RV-induced type I and type III IFN expression primarily depends on the activation of cytosolic dsRNA receptors such as MDA5 and RIG-I and endosomal dsRNA receptor TLR3^18–20^. Both MDA5 and RIG-I upon ligation to dsRNA interact with mitochondrial antiviral signaling protein (MAVS), which leads to MAVS activation and subsequent phosphorylation of IRF3 and the expression of type I and type III IFNs^21^. Upon interaction with dsRNA receptors, MAVS forms large aggregates by self-association, and these aggregates are highly potent in activating IRF3^22,23^. Oxidative stress by itself can induce MAVS aggregation independent of dsRNA receptors^24^ and such aggregation can potentially inhibit IFN responses to subsequent viral infection. However, the contribution of FOXO3a in preventing mitochondrial oxidative stress or MAVS aggregation is not known.

FOXO3a also plays a critical role in maintaining homeostasis by preventing autoinflammation in several organs via inhibition of NF-κB activation^25^. Further, FOXO3a expression was demonstrated to be reduced in the lungs of patients with chronic obstructive pulmonary disease^7,8^. In mice exposed to cigarette smoke, FOXO3a was shown to reduce lung inflammation via attenuating oxidative stress^8^. Previously, we demonstrated that expression of FOXO3a negatively correlates with IL-8 production in airway epithelial cells obtained from patients with chronic obstructive lung disease^7^. These observations indicate that in addition to modulating antiviral responses FOXO3a may also play a crucial role in maintaining homeostasis via inhibition of pro-inflammatory chemokine expression.

RV is responsible for the majority of common colds and stimulates robust expression of type I and type III IFNs and pro-inflammatory cytokines in airway epithelial cells *in vitro* and in mice *in vivo*^26^. In this study, we generated airway epithelium-specific Foxo3a knockout mice and a FOXO3a knockout human airway epithelial cell line and examined antiviral and pro-inflammatory responses of these *in vivo* and *in vitro* models to RV infection. We also elucidated one of the mechanisms by which FOXO3a contributes to aberrant host responses to RV infection.

## Results

### Knockdown of Foxo3a in the airway epithelium reduces Poly I:C -induced IFN responses, but not chemokine expression

**Figure 1:**
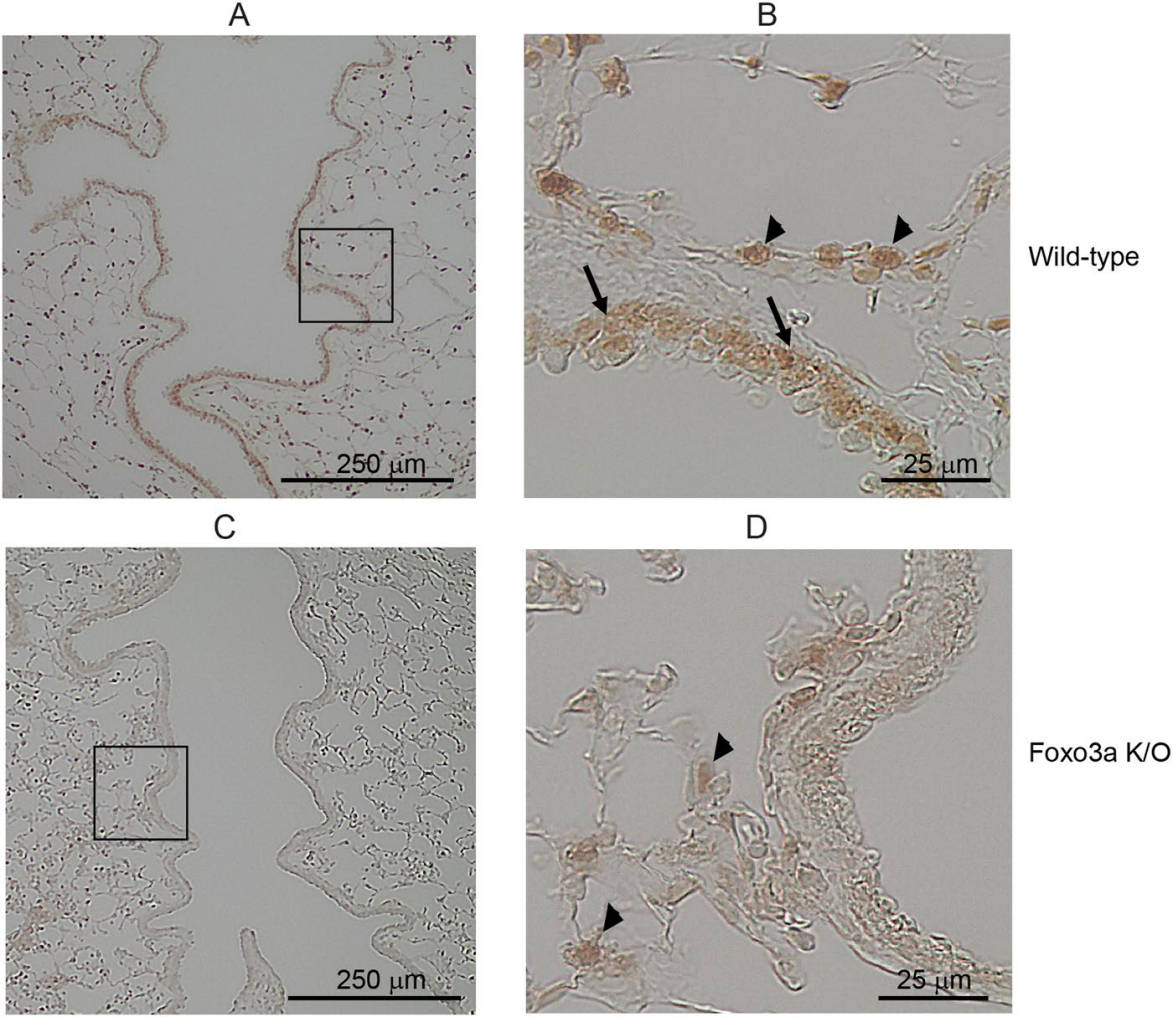
Confirmation of knockdown of Foxo3a in the airway epithelium of Foxo3a K/O mice. Lung sections from wild-type and Foxo3a K/O mice treated with tamoxifen were analyzed for expression of Foxo3a by immunohistochemistry. (**A**) Wild type mice immunostained with antibody to Foxo3a. (**B**) Represents magnified view of rectangle marked in panel A. (**C**) Foxo3a K/O mice immunostained with antibody to Foxo3a. (**D**) Represents magnified view of rectangle marked in panel C. Arrows in A, represent Foxo3a in airway epithelium and arrowheads in B and D represent Foxo3a in parenchymal cells.

Club cells are airway progenitor cells which self-renew and generate different cell types of airway epithelium including ciliated cells^27^. Therefore, we used Club cell-specific promoter to generate relatively stable airway epithelial-specific Foxo K/O mice. First, we confirmed the knockdown of Foxo3a in the airway epithelium of Foxo3a K/O mice 7 days after the last tamoxifen treatment. Wild-type mice showed the expression of Foxo3a in airway epithelial cells as well as in the parenchyma (Fig. 1A,B). In contrast, Foxo3a K/O mice showed expression of Foxo3a only in the parenchyma and not in the airway epithelium as expected (Fig. 1C,D). Lung sections from wild-type mice incubated with Foxo3a antibody absorbed against its own antigen showed no staining indicating the specificity of the antibody (data not shown). These results confirmed the knockdown of Foxo3a specifically in the airway epithelium of Foxo3a K/O mice.

**Figure 2:**
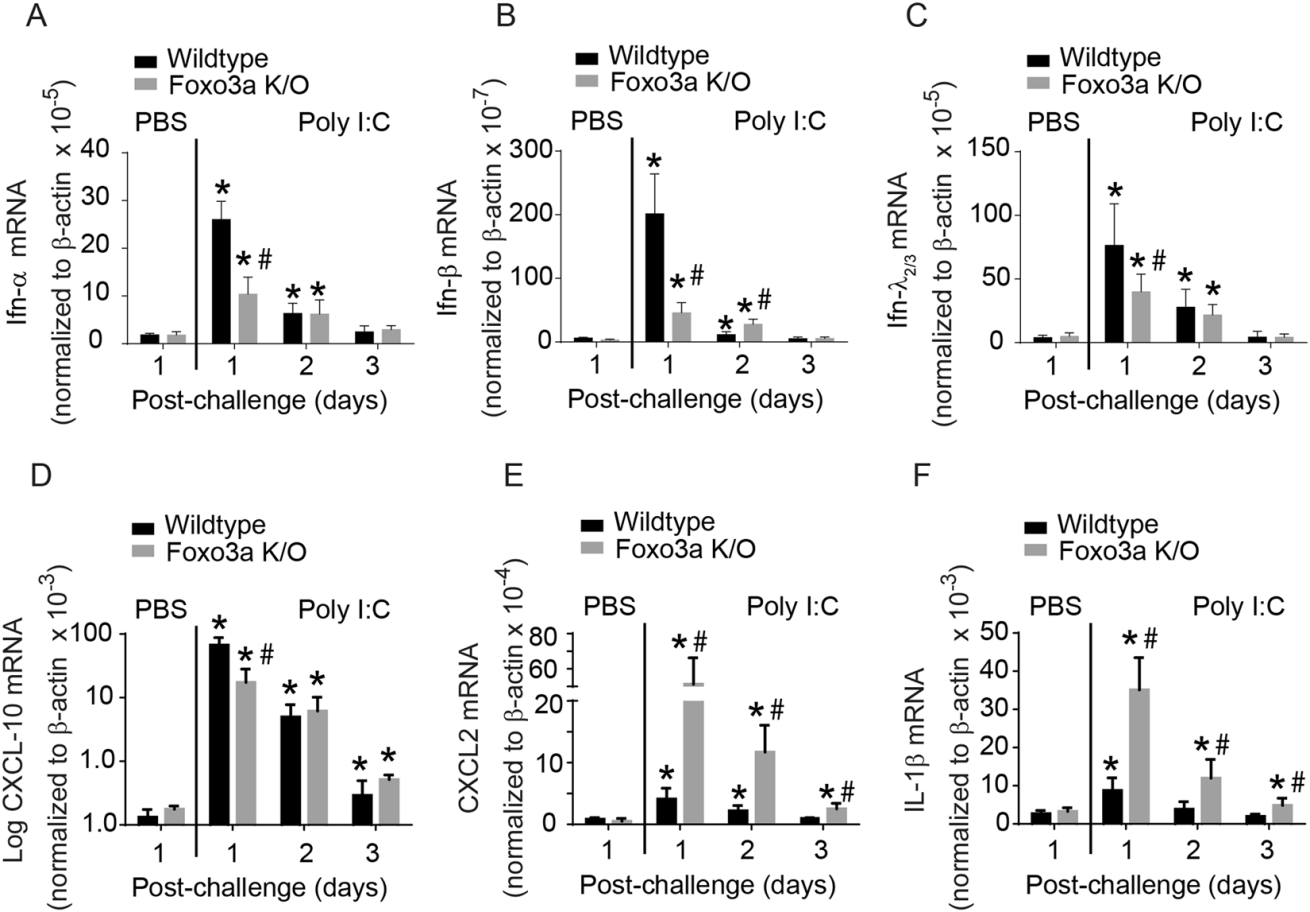
Innate immune responses of wild-type and Foxo3a K/O mice to Poly I:C challenge. Wild-type and Foxo3a K/O mice were challenged with Poly I:C by intranasal route and sacrificed 1, 2, or 3 days post-infection. (**A–F**) Total RNA was isolated from the lungs, reverse transcribed and subjected to probe-based qPCR. Data was normalized to housekeeping gene and represent mean and SD calculated from 3 independent experiments with 2 to 3 mice group to a total of 7–9 mice in each group. (*p ≤ 0.05, different from respective PBS-treated mice; ^#^p ≤ 0.05, different from similarly-challenged wild-type animals, one-way ANOVA).

All RNA viruses induce an IFN response via dsRNA intermediates generated during viral replication. Therefore in the initial experiment, we examined the contribution of FOXO3a in antiviral IFNs responses using dsRNA mimic Poly I:C. Wild-type and Foxo3a K/O mice were intranasally challenged with Poly I:C and the mRNA expression of antiviral *Ifns*, *Ifn-α*, *Ifn-β* and *Ifn-λ2/3* in total lung homogenates was determined by qPCR at 1, 2, and 3 days post-challenge. At 1 day post-challenge, Poly I:C-induced expression of all three *Ifn* genes and *CXCL-10* in both wild-type and Foxo3a K/O mice (Fig. 2A–D), but the expression levels of all three *Ifns* and *CXCL-10* were significantly lower in Foxo3a K/O than in wild-type mice. Interestingly, compared to wild-type, Foxo3a K/O mice showed higher expression of *Cxcl-2* and *Il-1β* (Fig. 2E,F) in response to Poly I:C challenge. Expression levels of all the measured cytokines returned to basal levels by 3 days after Poly I:C challenge in both types of mice. These results indicate that Foxo3a plays an important role in stimulating the expression of antiviral *Ifn*s, and limiting the pro-inflammatory cytokines in mice challenged with dsRNA mimic poly I:C.

### Foxo3a K/O mice show reduced antiviral IFN and CXCL-10 responses to RV infection

**Figure 3:**
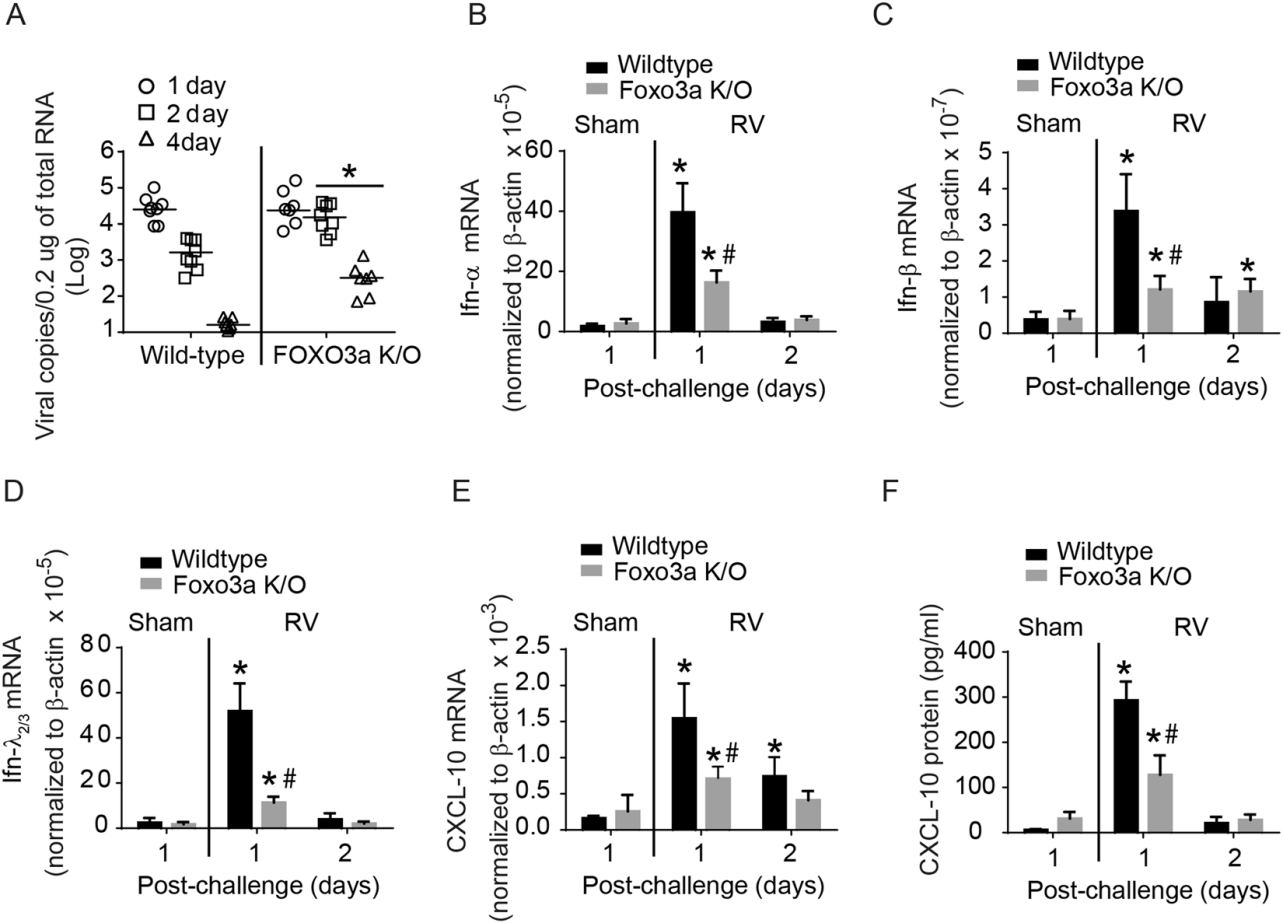
Antiviral responses of wild-type and Foxo3a K/O mice to RV infection. Wild-type and Foxo3a K/O mice were infected with RV or sham by intranasal route and sacrificed 1, 2 or 7 days post-infection. (**A**) Total RNA was isolated from the lungs and subjected to quantitative RT-qPCR to determine viral RNA. (*p ≤ 0.05, different from RV-infected wild-type mice). (**B–E**) cDNA was synthesized from total RNA isolated at 1 and 2 days post-infection and subjected to probe-based qPCR. Data in each panel was normalized to housekeeping gene and reported as mean and SD calculated from 3 independent experiments with 2 to 3 mice group to a total of 7–9 mice in each group. (**F**) Wild-type and Foxo3a K/O mice were infected with RV and sacrificed 1, or 2 days post-infection. BAL fluid was collected and CXCL-10 protein level was determined by multiplex Luminex ELISA. Data represent range with a median calculated from 6–8 mice per group. (*p ≤ 0.05, different from respective sham-infected animals; ^#^p ≤ 0.05, different from similarly-infected wild-type animals, one-way ANOVA).

RV, an RNA virus generates dsRNA as an intermediate during replication and thus stimulates IFNs, which play a crucial role in viral clearance. Replicating RV also stimulates CXCL-10, which attracts cytotoxic T cells to kill the infected cells. Therefore we examined whether Foxo3a K/O mice show defect in viral clearance. Wild-type and Foxo3a K/O mice were infected with RV or sham by intranasal route and viral load in the lungs was determined by measuring viral RNA, which is more sensitive. There was no significant difference in the viral load at 1 day post-infection between wild-type and Foxo3a K/O mice (Fig. 3A). However, at 2 and 4 days post-infection, Foxo3a K/O mice showed 1 to 2 logs higher viral load than wild-type mice. At 7 days post-infection, there was no detectable viral RNA in both types of mice (data not shown). To examine whether the defect in viral clearance is associated with attenuated antiviral responses, we assessed the mRNA expression of type I and III *Ifns*, and *Cxcl-10* at 1 and 2 days post-infection. These time points were chosen based on our previous report in which we demonstrated that RV-induced *Ifns* and *Cxcl-10* increase up to 24 h and then return to normal levels by 2 days post-infection^28^. Both wild-type and Foxo3a K/O mice showed expression of all three *Ifns* and *Cxcl-10* at 1 day post-RV infection, but the levels were significantly lower in Foxo3a K/O than in wild-type mice (Fig. 3B–E) as observed with poly I:C challenge. Levels of all three *Ifns* and *Cxcl-10* showed a reducing trend at 2 days post-infection in both Foxo3a K/O and wild-type mice. Protein level of CXCL-10 in bronchoalveolar lavage (BAL) fluid was also found to be higher in wild-type than in Foxo3a K/O mice following RV infection (Fig. 3F). Protein levels of IFN-α, IFN-β, IFN-λ_2_ in the BAL fluid were determined by ELISA and the levels of all three IFNs were below the detection limit (data not presented). These results indicate that Foxo3a K/O mice may have a defect in viral clearance, and this may be due to reduced type I and III *Ifns* and CXCL-10 expression.

### RV-infected Foxo3a mice show lung inflammation up to 4 days

**Figure 4:**
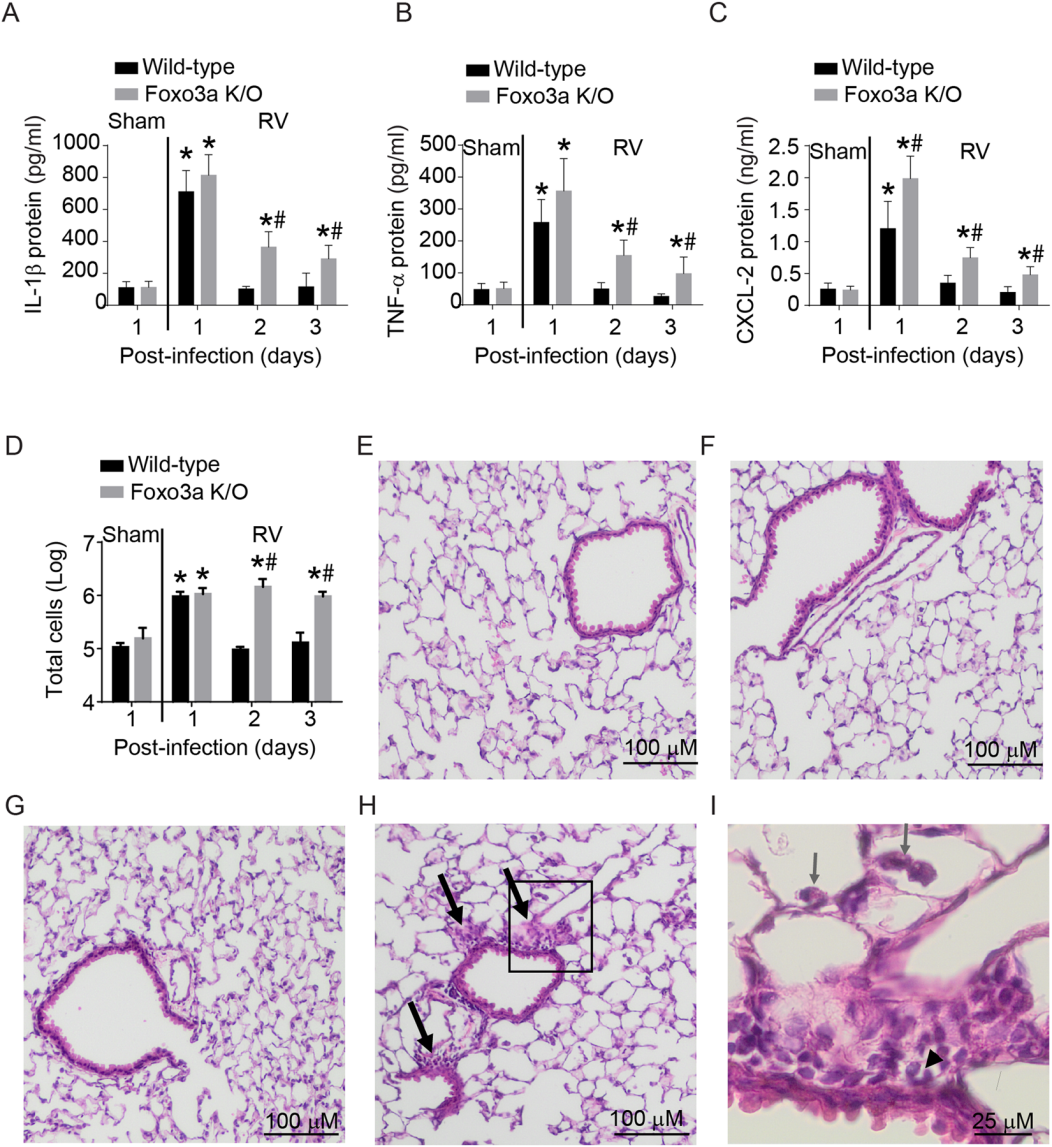
RV infection induces sustained lung inflammation in Foxo3a K/O mice. (**A–C**) Wild-type and Foxo3a K/O mice were infected with RV by intranasal route and sacrificed 1, 2, or 3 days post-infection. Sham-infected animals were sacrificed at 1 day post-infection. Lungs were lavaged and levels of cytokines were determined in BAL fluid by multiplex Luminex ELISA. (**D**) Total cells in the lavage were quantified by counting the cells. Data represent mean and SD calculated from 3 independent experiments with 2 to 3 mice group to a total of 7–9 mice in each group. (*p ≤ 0.05, different from respective sham-infected animals; ^#^p ≤ 0.05, different from similarly-infected wild-type animals, one-way ANOVA). (**E,F**) H & E sections of lung sections from wild-type animals infected with sham and RV respectively at 4 days post-infection. (**G,H**) H & E sections of lung sections from Foxo3a K/O animals infected with sham and RV respectively. Arrows represent areas of inflammation. (**I**) Magnified area marked (square) in panel H. Arrow and arrowheads respectively represent macrophages in alveolar space and neutrophils in a peribronchiolar area. Images are representative of 3 to 4 animals per group.

In addition to IFNs, RV also stimulates pro-inflammatory cytokines which may contribute to lung inflammation. Therefore we determined the protein levels of representative pro-inflammatory cytokines such as IL-1β, CXCL-2, and TNF-α, which we have shown to increase following RV infection in mice previously^28–30^. Protein levels of these pro-inflammatory cytokines were determined in BAL fluid by multiplex ELISA. Both wild-type and Foxo3a K/O mice showed increases in the protein levels of all three cytokines following RV infection (Fig. 4A–C). While all the cytokine levels returned to basal levels in wild-type mice by 2 days post-infection, Foxo3a mice showed sustained expression of IL-1β, CXCl-1, and TNF-α up to 3 days. Foxo3a K/O mice infected with RV also showed increase in the number of infiltrated inflammatory cells at 2 and 3 days post-infection as assessed by counting the total cells present in the BAL (Fig. 4D). Additionally, while wild type mice show normal lung morphology irrespective of infection (Fig. 4D,E), RV-infected Foxo3a k/O mice showed mild peribronchiolar inflammation at 3 days post-RV infection (Fig. 4F,H). Examining the inflamed area under high magnification indicated that accumulated inflammatory cells were primarily mononuclear cells, such as macrophages and lymphocytes with occasional neutrophils (Fig. 4G). These results indicate that Foxo3a in addition to stimulating optimal *Ifn* expression may also limit inflammatory responses to RV infection *in vivo*.

### FOXO3a deletion attenuates RV-induced IFN responses in human airway epithelial cells

**Figure 5:**
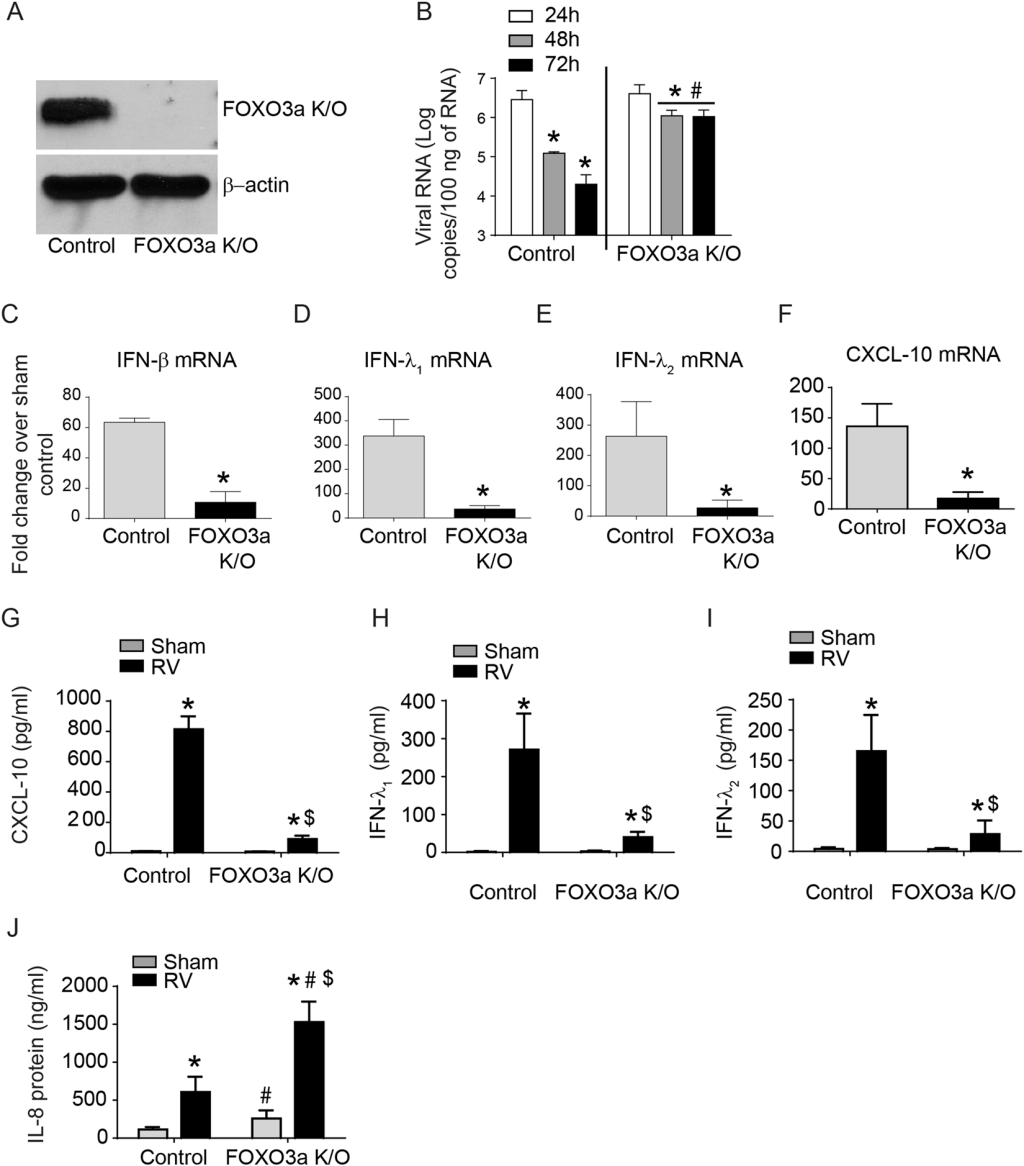
Responses of control and FOXO3a K/O airway epithelial cells to RV infection. (**A**) Western blot showing knockdown of FOXO3a in FOXO3a K/O cells. (**B**) Control vector or FOXO3a cells were infected with RV or sham and RNA was isolated at 24, 48 and 72 h post-infection and subjected to quantitative qPCR to determine viral load. Data represent mean and SD calculated from 5 independent experiments conducted in triplicates (*p ≤ 0.05, different from respective 24 h post-RV infected cells; ^#^p ≤ 0.05, different from control vector cells at 48 and 72 h post-infection, ANOVA). (**C–F**) Total RNA was isolated 16 h after RV infection and subjected to probe-based qPCR. Data in each panel was normalized to housekeeping gene and expressed as fold change over respective sham-infected cells (* p ≤ 0.05, different from RV-infected control cells, t test). (**G–J**) Basolateral medium was collected 24 h after sham or RV infection and protein levels of CXCL-10, IFN-λ1, IFN-λ2, and IL-8 were determined by ELISA (*p ≤ 0.05, different from respective sham-infected cultures; ^#^p ≤ 0.05, different from sham-infected control vector cells; ^$^p ≤ 0.05, different from RV-infected control vector cells, one-way ANOVA).

We then sought to understand the mechanisms by which FOXO3a contributes to RV-induced IFN expression in airway epithelial cells. We generated a stable FOXO3a K/O human bronchial epithelial cell line (BEAS-2B) (Fig. 5A), infected with sham or RV and examined for viral load and expression of antiviral cytokines, IFN-β, IFN-λ1, IFN-λ2 and CXCL-10 and pro-inflammatory cytokine IL-8. There was no difference in the viral load based on the viral RNA copy number at 24 h post-infection between control and FOXO3a K/O cells (Fig. 5B). However at 48 and 72 h post-infection, there was significantly more viral RNA in FOXO3a K/O cells than control cells. FOXO3a K/O cells showed attenuated mRNA expression of all three IFNs and CXCL-10 in response to RV infection compared to control cells (Fig. 5C–F) similar to that observed in Foxo3a K/O mice. This was not due to altered mRNA expression of IFNs in sham-infected cells (data not shown). Protein levels of RV-induced IFN-λ1, IFN-λ2, and CXCL-10 were also reduced in FOXO3a K/O cells compared to control cells (Fig. 5G–I). IFN-β protein levels were below the detection limit and therefore could not be determined (data not shown). In contrast to IFN and CXCL-10 expression, FOXO3a K/O cells showed more IL-8 under unstimulated (sham) conditions, which further increased after RV infection (Fig. 5J). Together these results were very similar to that observed *in vivo* indicating the suitability of this *in vitro* model to determine the mechanisms by which FOXO3a contributes to RV-induced IFN responses.

### FOXO3a knockout cells show attenuated dsRNA receptor signaling axis

**Figure 6:**
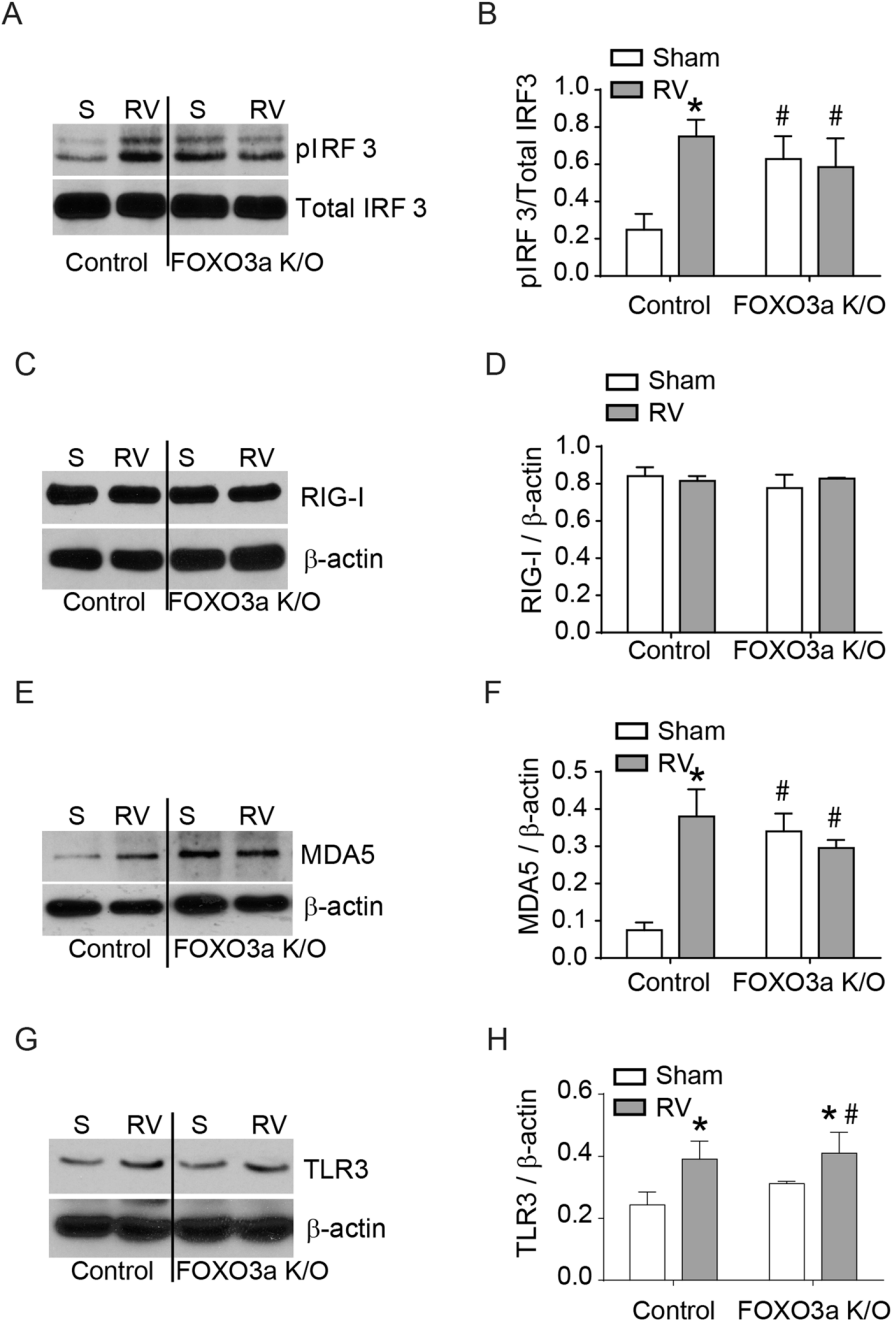
FOXO3a K/O cells show activation of MDA5 signaling pathway under basal conditions. Control vector and FOXO3a K/O airway epithelial cells were infected with sham or RV, and total protein was harvested at 4 h post-infection. (**A,C,E,G**) Equal amount of protein was subjected to Western blot analysis. Images are representative of 4 independent experiments. (**B,D,F,H**) Band intensities were determined by using Image J and expressed as a ratio of phospho- to total protein or total protein to β-actin. Data represent mean and SD calculated from 4 independent experiments (*p ≤ 0.05, different from respective sham-infected cultures; ^#^p ≤ 0.05, different from sham-infected control vector cells, ANOVA).

Previously, it has been demonstrated that RV induces IFN expression via activation of dsRNA receptors MDA5, RIG-I and TLR3^19^. Ligation of dsRNA to these receptors induces activation of IRF3^21,31^, therefore, we examined RV-induced phosphorylation of IRF3 in FOXO3a K/O and control vector cells. Control cells showed an increase in the phosphorylation of IRF3 after RV infection as observed previously^19^. In contrast, FOXO3a K/O cells showed increased phosphorylation of IRF3 under unstimulated conditions which did not increase further following RV infection (Fig. 6A,B). These results indicate that the deletion of FOXO3a may activate IRF3 and this may inhibit IRF3 activation to subsequent viral infection.

To examine whether the observed defect in IRF3 phosphorylation following RV infection is due to altered expression of dsRNA receptors in FOXO3a K/O cells, we performed Western blot analysis. RIG-I, which is constitutively expressed in bronchial epithelial cells was expressed equally in both control and FOXO3a K/O cells and did not increase following RV infection in both cell types (Fig. 6C,D). In contrast, MDA5, which is induced by viral infection, increased following RV infection in control, but not in FOXO3a K/O cells. Surprisingly FOXO3a K/O cells showed upregulation in the expression of MDA5 at basal levels (Fig. 6E,F). There was no difference in the expression of TLR3 between control and FOXO3a K/O cells (6G and 6H). There was also no difference in the expression of either MAVS or TRIF, adaptor proteins necessary for IRF3 activation between control and FOXO3a K/O cells (data not shown). MAVS and TRIF are adaptor proteins for RIG-I/MDA5 and TLR3 respectively.

### Inhibition of oxidative stress restores RV-induced IFN expression in FOXO3a K/O cells

**Figure 7:**
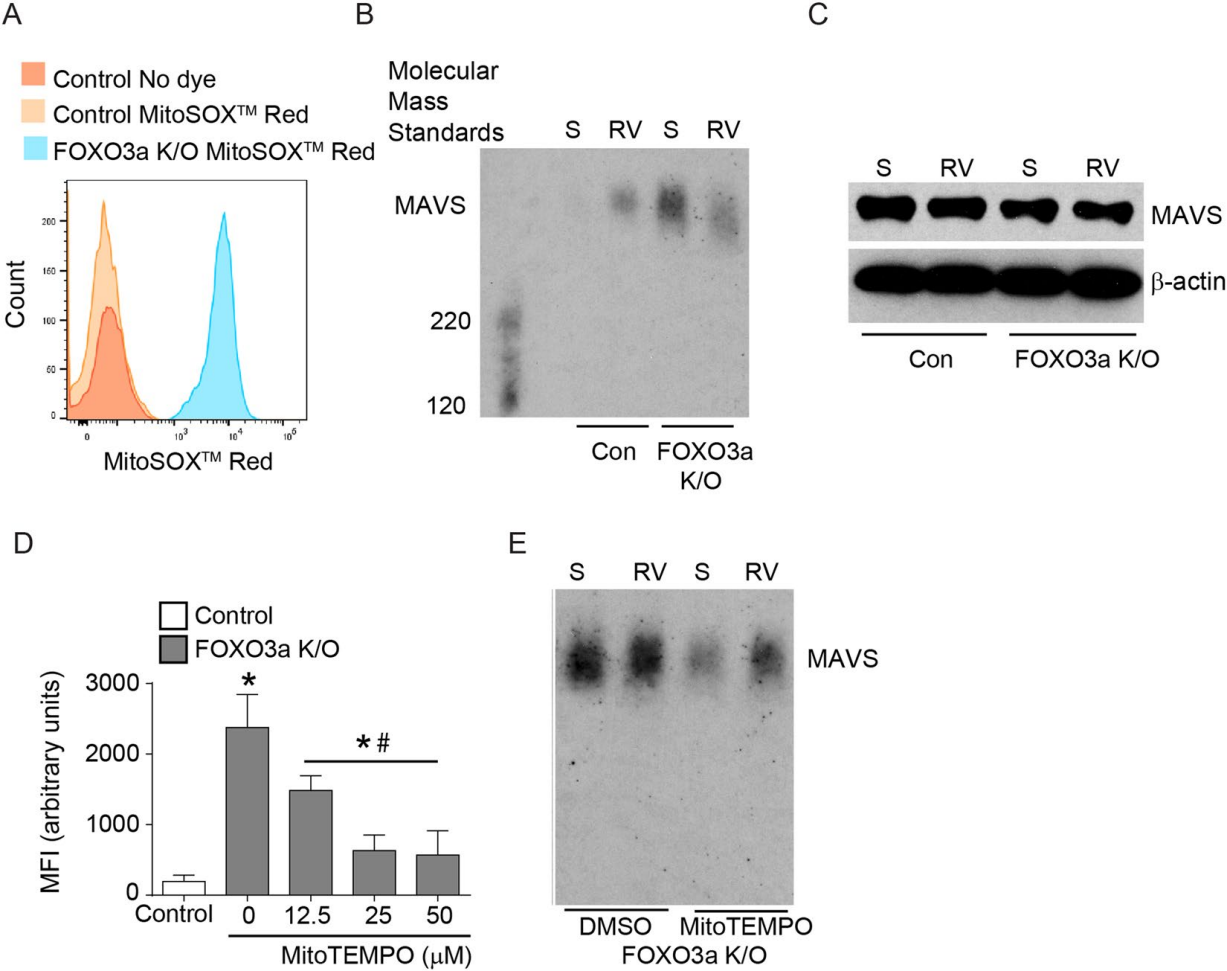
Oxidative stress and MAVS polymerization is increased in FOXO3a K/O airway epithelial cells under basal conditions. (**A**) Control vector and FOXO3a K/O cells grown to 90% confluence were loaded with MitoSOX Red, washed and analyzed by flow cytometry. Histogram is a representative of 3 independent experiments performed in triplicates. (**B,C**) Control vector and FOXO3a K/O cells were infected with sham or RV, incubated for 4 h, and crude mictochondrial proteins or total proteins were isolated. Crude mitochondrial extract was subjected to SDD-AGE to assess the oligomerization of MAVS (**B**), and total protein was subjected to SDS-PAGE to assess total MAVS (**C**). (**D**) FOXO3a K/O cells were treated with MitoTEMPO for 3 h, loaded with MitoSOX Red and analyzed by flow cytometry. MitoSOX Red loaded control vector cells were used as negative controls. Data represent mean and SD calculated from 3 independent experiments performed in triplicates (*p ≤ 0.05, different from control vector cells; ^#^p ≤ 0.05, different from DMSO treated FOXO3a K/O cells, ANOVA). (**E**) FOXO3a K/O cells pretreated with DMSO or 25 μM MitoTEMPO were infected with sham or RV and incubated for 4 h without MitoTEMPO. Mitochondrial crude extract was subjected to SDD-AGE to assess MAVS oligomerization. Images in panels (B, C, and E) are representative of 3 independent experiments.

Activation of dsRNA receptors RIG-I/MDA5 by dsRNA induces change in MAVS conformation from monomeric to aggregated (active) form^22,23^. FOXO3a regulates antioxidant genes^14–16^ and therefore knockdown of FOXO3a can induce excessive oxidative stress and this can potentially alter MAVS conformation^24^. Therefore, we determined the oxidative stress and aggregation of MAVS in control and FOXO3a K/O cells by flow cytometry and semidenaturing detergent agarose gel electrophoresis (SDD-AGE), respectively. FOXO3a K/O cells showed increased oxidative stress compared to control cells under unstimulated conditions (Fig. 7A). While control cells showed aggregation of MAVS only after RV infection (Fig. 7B), FOXO3a K/O cells showed MAVS aggregation under unstimulated conditions (sham infection), which did not increase after RV infection. There was no difference in the total protein levels of MAVS between control and FOXO3A K/O cells irrespective of RV infection (Fig. 7C). These results indicate that increased oxidative stress in FOXO3a K/O cells may induce conformational changes in MAVS, and this may inhibit the interaction of activated dsRNA receptors with MAVS following RV infection.

Since MAVS is primarily expressed in mitochondria, we examined whether treatment with MitoTEMPO, a mitochondrial-specific antioxidant reduces oxidative stress and changes MAVS conformation in FOXO3a K/O cells. FOXO3a K/O cells showed significantly higher oxidative stress than control cells as observed above (Fig. 7A), which decreased after treatment with MitoTEMPO in a dose-dependent manner showing a maximum reduction at 25 μM (Fig. 7D). Therefore, in subsequent experiments we pretreated cells with 25 μM MitoTEMPO. Pretreatment with MitoTEMPO reduced MAVS aggregation in FOXO3a K/O cells (Fig. 7E). Intriguingly, FOXO3a K/O cells pretreated with MitoTEMPO showed increased MAVS aggregation similar to control cells only after RV infection. These results suggest that inhibition of oxidative stress reduces MAVS aggregation in unstimulated FOXO3a K/O cells and this may improve RV-induced dsRNA receptor signaling via interaction with MAVS.

**Figure 8:**
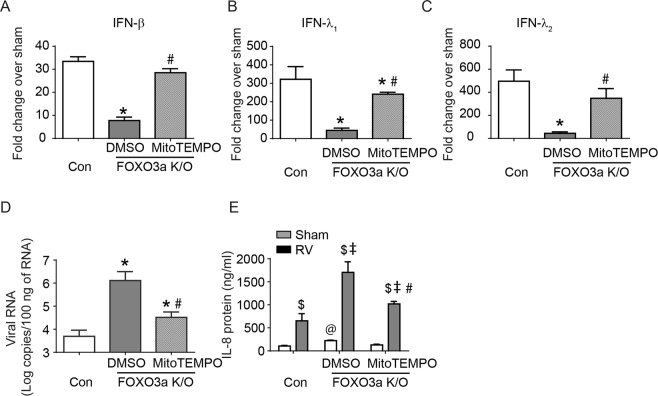
Pretreatment with MitoTEMPO corrects innate immune responses to RV infection in FOXO3a K/O cells. FOXO3a K/O cells pretreated with DMSO or 25 μM MitoTEMPO, and control vector cells were infected with sham or RV and incubated for 16 h without MitoTEMPO. (**A–D**) Total RNA was isolated and subjected to probe-based qPCR to determine the mRNA expression levels of IFNs or quantitative probe-based assay to assess viral RNA. (**A–C**) mRNA expression levels were normalized to housekeeping gene and expressed as fold increase over respective sham-infected cultures. (**D**) Viral RNA was quantified 48 h after RV infection and data were expressed as number of viral RNA copies/100 ng of total RNA (*p ≤ 0.05, different from RV-infected control cells; ^#^p ≤ 0.05, different from DMSO treated FOXO3a K/O cells, ANOVA). (**E**) Cell culture medium was harvested at 16 h post-infection and used for assessing protein levels of IL-8 by ELISA. Data represent mean and SD calculated from 3 independent experiments performed in duplicates or triplicates. (^$^p ≤ 0.05, different from respective sham-infected cultures; ^@^p ≤ 0.05, different from sham-infected control vector cells; ^‡^p ≤ 0.05, different from RV-infected control vector cells; ^#^p ≤ 0.05, different from DMSO treated FOXO3a K/O cells, ANOVA).

To examine whether MitoTEMPO also corrects IFN responses to RV infection, FOXO3a K/O cells were pretreated with 25 μM MitoTEMPO overnight, cells were washed once and then infected with sham or RV and mRNA levels of IFNs was measured after 16 hours. FOXO3a K/O cells pretreated with MitoTEMPO showed a significant increase in RV-induced IFNs expression compared to DMSO pretreated cells (Fig. 8A–C). Besides MitoTEMPO pretreated FOXO3a K/O cells also showed less viral RNA compared to DMSO treated cells (Fig. 8D). Interestingly, MitoTEMPO pretreatment also reduced RV-stimulated IL-8 levels (Fig. 8E).

**Figure 9:**
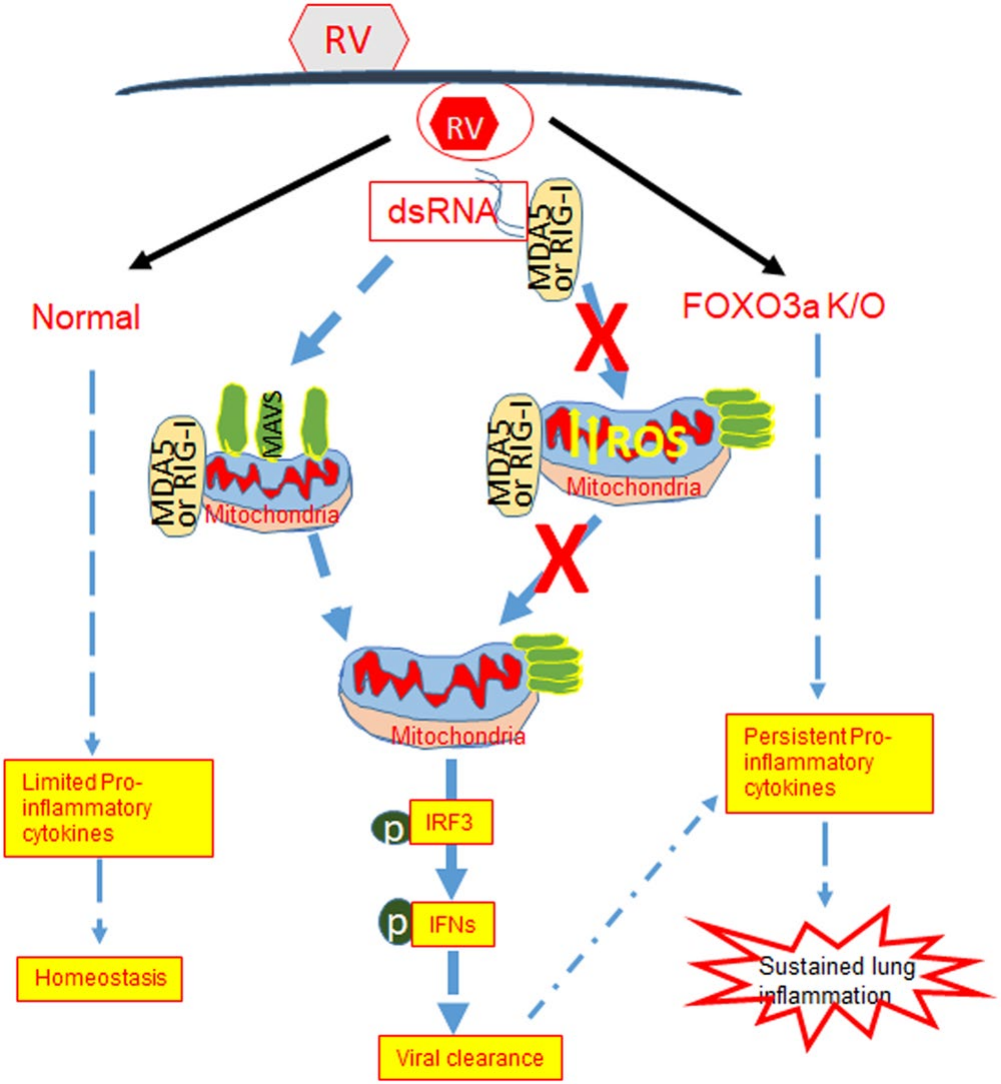
Overview of the results. Deletion of FOXO3a in airway epithelial cells increases mitochondrial ROS leading to MAVS conformational change. This in turn attenuates virus-stimulated dsRNA receptor, IRF3 activation and antiviral IFN expression resulting in viral persistence. Deletion of FOXO3a further enhances RV-induced pro-inflammatory cytokines either directly or by increasing viral load leading to sustained lung inflammation.

Taken together these results indicate that FOXO3a may be required for preventing oxidative stress in mitochondria, thus maintaining the conformation of MAVS. The latter is required for dsRNA receptor activation following RV infection to stimulate IFN responses and optimal viral clearance. Furthermore, our data indicate that FOXO3a may also play a role in limiting RV-induced pro-inflammatory cytokine responses either directly or by promoting viral clearance thus preventing sustained lung inflammation (Fig. 9).

## Discussion

FOXO3a plays an important role in the regulation of inflammation by limiting cytokine production, thus maintaining homeostasis. Here we demonstrate a unique role for FOXO3a in the regulation of antiviral IFN responses to RV infection in airway epithelial cells both *in vitro* and *in vivo*. Furthermore, we show that FOXO3a is essential for preventing excessive mitochondrial oxidative stress, which can potentially alter the conformation of MAVS, thus impairing antiviral IFN responses.

Previously, FOXO3a was shown to play a role in stimulating the expression of pro-inflammatory cytokines including IL-8, CXCL-10, IL-6 and TNF-α in response to Poly I:C challenge in the airway epithelial cells *in vitro*^9^. Poly I:C is a dsRNA mimetic and is usually used as a surrogate for dsRNA generated during viral replication. In this study, while FOXO3a positively regulated RV-induced antiviral IFN expression, it negatively regulated RV-stimulated pro-inflammatory responses indicating opposing role of FOXO3a in regulation of antiviral and pro-inflammatory cytokine responses. RV-induced IFNs or CXCL-10 primarily depends on the recognition of dsRNA intermediate generated during viral replication by dsRNA receptors, such as  cytoplasmic receptors MDA5 and RIG-I, and endosomal receptor TLR3^18–20^. IRF3 is a common transcription factor that gets phosphorylated/activated downstream of ligation of dsRNA to its receptors^21^. Previously we have demonstrated that RV induces MDA5 expression, and knockdown of MDA5 inhibits RV-induced IRF3 activation, as well as IFN responses significantly indicating that RV stimulates IFN expression primarily via MDA5^19^. Therefore it is possible that the deletion of FOXO3a may inhibit RV-induced IFNs by affecting the expression or downstream signaling of dsRNA receptors. There was no difference in the expression of RIG-I or TLR3 between control and FOXO3a K/O cells prior to or after RV infection. In contrast, unlike control, FOXO3a K/O cells did not show an increase in either the expression of MDA5 or phosphorylation of IRF3. Intriguingly, FOXO3a K/O cells showed higher expression of MDA5 under unstimulated conditions, and this was associated with increased IRF3 phosphorylation, but not IFN expression. These observations indicate that MDA5 receptor signaling may be already active in FOXO3a K/O cells leading to the desensitization of this signaling pathway. This may potentially attenuate antiviral IFN responses to subsequent viral infection.

Previously, we have shown that airway epithelial cells under oxidative stress show attenuated IFN response to RV infection and it was due to inhibition of MDA5 signaling^17^. Since FOXO3a regulates expression of phase II antioxidant genes, the absence of this transcription factor may induce oxidative stress in the cells^32^. Consistent with this notion, we found that FOXO3a K/O cells show enhanced oxidative stress. Pretreatment with MitoTEMPO, which neutralizes mitochondrial ROS reduced oxidative stress and restored IFN responses to subsequent RV infection indicating that the mitochondrial oxidative stress in FOXO3a K/O cells may contribute to attenuated dsRNA signaling.

MAVS when activated via dsRNA receptors forms aggregates on the mitochondria which then leads to recruitment and activation of IRF3^22,23^. Cells under oxidative stress show activation of MAVS signaling and IFN expression independent of dsRNA receptors and causes inflammation in autoimmune disease, Lupus erythematous^24^. FOXO3a K/O cells showed aggregation of MAVS under unstimulated conditions indicating activation of MAVS and this probably explains the observed increase in the IRF3 phosphorylation in these cells. Despite activation of MAVS and IRF3, FOXO3a K/O cells did not show increased expression of IFNs under unstimulated conditions. This may be due to the upregulation of endogenous negative regulators of IRF3, such as tripartite motif-containing protein (TRIM)26, protein phosphatase 2A, receptor of activated protein kinase (RACK)1, ubiquitin E3 ligase RAUL^33–35^. Although negative regulation of IRF3 signaling is necessary to prevent persistent expression of unwanted cytokines, it may attenuate IFN response to subsequent viral infection.

In addition to inducing MAVS activation independent of dsRNA receptor signaling, oxidative stress may also enhance the expression of MDA5 in the absence of viral infection. In the resting state, MDA5 is expressed at very low levels, but upon viral infection, its expression is strongly induced by type I interferons via activation of JAK/STAT signaling pathway^36^. Recently oncostatin, a member of the IL-6 family was shown to induce MDA5 via activation of JAK/STAT1 signaling and provide protection against subsequent virus infection^37^. Persistent oxidative stress can induce pro-inflammatory cytokines such as IL-8, IL-6 or IL-6 family members. Since unstimulated FOXO3a K/O cells show oxidative stress-dependent IL-8 but not type I IFNs expression in airway epithelial cells, we speculate that IL-8 may enhance the expression of MDA5 in these cells via activation of JAK/STAT signaling pathway^38^. Studies examining the mechanisms underlying the upregulated expression of MDA5 in FOXO3a K/O cells are currently under progress and will be the topic for future publication.

In the present study we found that in addition to promoting IFN responses, FOXO3a was also required for limiting expression of RV-induced pro-inflammatory cytokines both *in vitro* and *in vivo*. The observed persistent RV-induced pro-inflammatory responses may be simply due to increased viral load in the absence of FOXO3a. However this does not explain the enhanced expression of pro-inflammatory cytokines in the initial stages of infection (24 h post-infection), when both wild-type and Foxo3a K/O mice show similar viral load or when mice were challenged with poly I:C. We speculate that FOXO3a which negatively regulates NF-ĸB^25^ may play a role in limiting RV-stimulated expression of pro-inflammatory cytokines, which primarily depend on the activation of NF-ĸB^39,40^. In contrast to our observations, in previous studies, FOXO3a knockdown by transient transfection with FOXO3a siRNA was shown to inhibit Poly I:C-stimulated IL-8, IL-6, TNF-α in airway epithelial cells *in vitro*^9^. At present, we don’t know the reasons for the observed discrepancy, but it may depend on type of Poly I:C used. We used high molecular weight Poly I:C to mimic long dsRNA generated during RV replication and long dsRNA specifically stimulates MDA5.

Taken together, our data demonstrate that the absence of FOXO3a in the airway epithelial cells alters the antiviral innate immune responses to RV infection, leading to the suppression of antiviral type I and type III IFNs production by decreasing activation of MDA5/MAVS signaling. An impaired early epithelial-initiated antiviral response to RV infection may enhance viral replication and persistence *in vivo*. The absence of FOXO3a may also increase the expression of pro-inflammatory cytokines in response to RV infection leading to sustained lung inflammation. In conclusion, our results indicate that FOXO3a plays a crucial role in viral clearance and limiting lung inflammation by modulating early innate immune responses of airway epithelial cells following viral infection.

## Methods

### Generation of Club cell-specific FOXO3a K/O mice

*Scgb1a1-*CreER^TM^ and *Foxo3*^*tm1Rdp*^/J were purchased from Jackson Laboratory (Bar Harbor, Maine). To generate Club cell-specific Foxo3a K/O (*Scgb1a1-Fox3a*^−/−^) mice, homozygous *Scgb1a1-*CreER^TM^ male was bred with homozygous Foxo3^*tm1Rdp/J*^ female. Heterozygotes from this generation were bred to obtain second-generation males which are homozygous for *Scgb1a1-*CreER^TM+/+^ and heterozygous for FOXO3a^−/+^. These males were backcrossed with homozygous female *Foxo3*^tm1Rdp^/J mice to obtain *Scgb1a1-Fox3a*^−/−^ and *Scgb1a1-Fox3a*^*+/−*^. *Scgb1a1-Fox3a*^−/−^ (Foxo3a K/O) mice were treated with tamoxifen for five consecutive days to knockdown Foxo3a between 4–5 weeks of age and mice were used in the experiments one week after completion of tamoxifen treatment. Similarly treated age-matched littermate *Scgb1a1-Fox3a*^+*/*−^ (wild-type) served as controls. Both male and female mice were used in this study since there was no major difference in response to RV infection between males and females. All the studies were approved by the Institutional Animal Care and User Committee at Temple University, Philadelphia. All methods were performed in accordance with the relevant guidelines and regulations.

### Stable FOXO3a K/O airway epithelial cell line

BEAS-2B cells were transduced with GIPZ Lentiviral vector (Dharmacon, Chicago, IL) expressing either FOXO3a shRNA or non-targeting shRNA and cultured in the presence of 2 μg/ml puromycin to select for transduced cells. The cell line showing FOXO3a knockdown as determined by protein expression was expanded and stored. The FOXO3a knockout cells were maintained in BEGM containing 2 μg/ml puromycin in all the experiments.

### Poly I:C challenge

Mice were challenged with 25 μg of high molecular weight poly I:C (Invivogen, San Diego, CA) dissolved in 50 μl of endotoxin free PBS (Thermofisher Scientific, Waltham, MA) or equal volume of PBS by the intranasal route as described previously^28^ and sacrificed at 1, 2, or 3 days post-infection by asphyxiation. Lungs were harvested and processed for total RNA isolation.

### Rhinovirus stock and infection

RV1B was purchased from ATCC (Manassas, VA). Viral stocks for infection were prepared by infecting H1HeLa cells with RV1B and subjecting HeLa cell supernatants to ultrafiltration as described previously^17,41^. Similarly, concentrated and purified cell supernatants from uninfected H1HeLa cells were used as sham controls. Mice were infected with RV or equal volume of sham by the intranasal route as described previously^28^ and sacrificed at 1, 2, 3, 4 or 7 days post-infection by asphyxiation. Lungs were harvested and processed for total RNA isolation or perfused via the right ventricle to remove blood cells, and bronchoalveolar lavage (BAL) performed with 1 ml PBS containing protease inhibitors. BAL was centrifuged and the supernatant was stored frozen until analyzed by multiplex ELISA. The pellet was suspended in 1 ml PBS and total number of cells was determined. In some experiments lungs were perfused with PBS, inflation fixed with 4% paraformaldehyde and embedded in paraffin.

Control or FOXO3a K/O cells were infected with sham or RV at multiplicity of infection (MOI) of 1 or equal volume of sham and incubated for 90 min at 33 °C. The infection medium was replaced with fresh medium and incubation continued at 33 °C for up to 72 h.

### Histology and Immunohistochemistry

Mouse lung sections were deparaffinized, hydrated and stained with hematoxylin and eosin. For immunohistochemistry, deparaffinized and hydrated lung sections were treated with boiling antigen retrieval buffer (0.01 M citrate buffer, pH 6.0) for 10 minutes. Slides were then washed in PBS, treated with 3% hydrogen peroxidase to block endogenous peroxidase activity, blocked with 5% normal goat serum diluted in PBS containing 0.3% Triron X-100 and then incubated with Foxo3a Rabbit monoclonal antibody (Cell Signaling Technology, Danvers, MA). The bound antibody was detected by using Immunohistochemistry Application Solutions Kit (Rabbit) (Cell Signaling Technology). Lung sections incubated with Foxo3a antibody absorbed against its antigen served as negative control. Slides were observed and photographed with a light microscope equipped with a CCD camera.

### Flow Cytometry

After relevant treatment, control and FOXO3a cells were washed with HBSS, loaded with 5 ug/ml MitoSOX Red (ThermoFisher Scientific) diluted in HBSS, and incubated for 20 min. Cells were washed with HBSS, harvested using enzyme-free cell dissociation medium (ThermoFisher Scientific), and immediately analyzed by flow cytometry. BEAS-2B cells treated with 100 μM hydrogen peroxide for 15 minutes served as positive control. Control cells and control cells loaded with MitoSOX Red served as negative controls.

### Western blot analysis

After relevant treatment, cells were washed with cold PBS and lysed in RIPA buffer containing protease and phosphatase inhibitors. Equal amount of protein was subjected to sodium dodecyl sulphate (SDS) polyacrylamide gel electrophoresis under reducing conditions, proteins transferred to nitrocellulose membrane and subjected to Western blot analysis with antibodies to FOXO3a, MDA5, TLR3, MAVS (Santa Cruz Biotechnology, Santa Cruz, CA), total and phospho-IRF3 (Cell Signaling), or β-actin (Sigma Aldrich, Saint Louis, MO). Specific bands were quantified by densitometry using NIH Image J, and the results expressed as fold change over β-actin or respective total protein. To determine MAVS aggregation, we performed semidenaturing detergent agarose gel electrophoresis (SDD-AGE) under non-reducing conditions as described previously using 1.5% agarose^22^ with some modifications. Briefly, mitochondrial crude extracts were resuspended in sample buffer (0.5 X TBE containing 10% glycerol, 2% SDS and 0.00025% bromophenol blue) and loaded on horizontal 1.5% agarose gel and the gel was run at 50 volts at 4 °C overnight. The separated proteins were passively transferred to immobilon overnight and subjected to Western blot analysis with an antibody to MAVS.

### Real-time PCR

After relevant treatment, total RNA was isolated from airway epithelial cells or mouse lungs and the expression of CXCL-10, IFN-β, IFN-λ1, IFN-λ3 in airway epithelial cells and Cxcl-10, Ifn-β, Cxcl-2, Il-1β in mouse lungs was determined by using gene-specific primers and normalized to housekeeping gene, glyceraldehyde 3-phosphate dehydrogenase (G3PDH) or β-actin^42^. PrimeTime qPCR Probe assays for all the above genes were purchased from Integrated DNA Technologies (Coralville, IA). Viral RNA copy number was determined by quantitative qPCR as described previously^26,28^.

### Elisa

Custom multiplex Luminex assays were purchased from R & D systems (Minneapolis, MN) and the levels of cytokines and chemokines in the BAL supernatants were determined following the manufacturer’s instructions. Duoset IL-8 ELISA (R & D systems), IFN-λ_1_ and IFN-λ_2_ ELISA kits (ThermoFisher Scientific) were used for the determination of IL-8, IFN-λ_1_ and IFN-λ_2_ protein levels in the cell culture medium.

### Statistical analysis

SigmaStat 4.0 (Systat Software, Inc., San Jose, CA) was used to perform statistical analysis of all the data. Statistical significance for normally distributed data was assessed by unpaired student T test (for comparisons between 2 groups) or by analysis of variance (ANOVA) with Tukey-Kramer post hoc test (for comparisons between 3 or more groups). If the data was not normally distributed, we used non-parametric tests such as the Wilcoxon Rank Sum test to compare between 2 groups, and ANOVA on ranks with the Kruskal-Wallis H test for comparing 3 or more groups. A p value of ≤0.05 was considered as statistically significant.
